# Muscle–Bone Crosstalk: Emerging Opportunities for Novel Therapeutic Approaches to Treat Musculoskeletal Pathologies

**DOI:** 10.3390/biomedicines5040062

**Published:** 2017-10-24

**Authors:** Delphine B. Maurel, Katharina Jähn, Nuria Lara-Castillo

**Affiliations:** 1 Pharmaceutical Sciences Department, Université de Bordeaux, BioTis, INSERM Unité 1026, 146 Rue Léo Saignat, 33076 Bordeaux, France; 2 Department for Osteology and Biomechanics, University Medical Center Hamburg-Eppendorf, 20246 Hamburg, Germany; k.jaehn@uke.de; 3 Department of Oral and Craniofacial Sciences, School of Dentistry, University of Missouri- Kansas City, 650 E 25th St, Kansas City, MO 64108, USA; laran@umkc.edu

**Keywords:** muscle, bone, crosstalk, biomechanical, hormones, therapeutics

## Abstract

Osteoporosis and sarcopenia are age-related musculoskeletal pathologies that often develop in parallel. Osteoporosis is characterized by a reduced bone mass and an increased fracture risk. Sarcopenia describes muscle wasting with an increasing risk of injuries due to falls. The medical treatment of both diseases costs billions in health care per year. With the impact on public health and economy, and considering the increasing life expectancy of populations, more efficient treatment regimens are sought. The biomechanical interaction between both tissues with muscle acting on bone is well established. Recently, both tissues were also determined as secretory endocrine organs affecting the function of one another. New exciting discoveries on this front are made each year, with novel signaling molecules being discovered and potential controversies being described. While this review does not claim completeness, it will summarize the current knowledge on both the biomechanical and the biochemical link between muscle and bone. The review will highlight the known secreted molecules by both tissues affecting the other and finish with an outlook on novel therapeutics that could emerge from these discoveries.

## 1. Introduction

**Figure 1:**
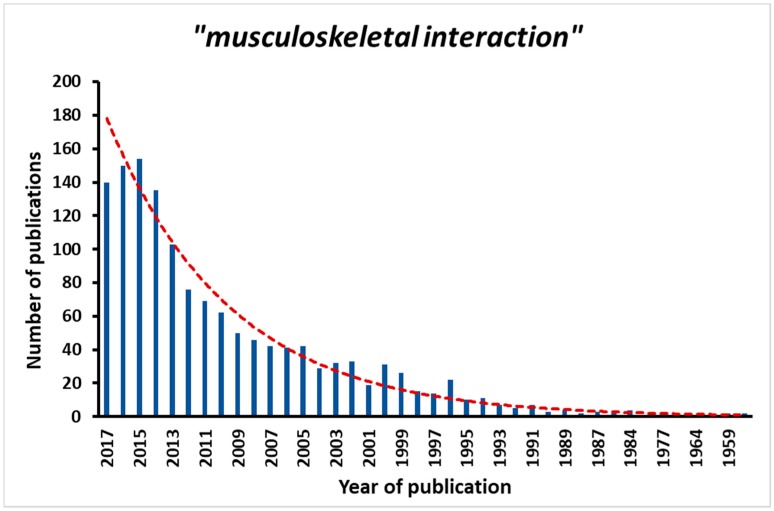
Using the search term “musculoskeletal interaction”, this graph demonstrated the increase in published papers in recent years with regard to the topic. The search has been made using PubMed, in September 2017.

Muscle and bone interactions are a current focus of research studies with a steady increase in the number of related publications since the early 2000s (Figure 1). Both the biomechanical and the biochemical interaction in the musculoskeletal unit appear of regulatory importance to enable tissue function. It is thought that skeletal muscle and the long bones grow together early in life, and are maintained and adapted to fit the metabolic and mechanical needs in healthy adults. However, as a functional unit, both tissues are also found to deteriorate together with disuse, disease or even with the process of aging. Aging, defined as the loss of tissue function over time, leads to a decline in both muscle and bone strength [1]. Age-related musculoskeletal degenerations are not only linked to a reduction in overall mobility and therefore lower the patient’s quality of life, but also to an increase in the risk of falls and concurrent fractures expanding the musculoskeletal burden even further. Fragility fractures in the elderly are correlated with higher morbidity and mortality [2]. With our demographic development and the desire to extend the overall life expectancy, osteoporosis and sarcopenia will affect millions of people in the near future [3]. The concurrent link of muscle and bone tissue quality suggests a huge pharmaceutical potential for efficient treatment regimens that act on both tissues simultaneously.

During the growth period, muscle and bone grow in proportion to one another. This phenomenon has been at the basis of the biomechanical interaction theory, where bone adapts to muscle forces during development [4]. In addition, the effects of physical activity, disuse and the aging-related diseases of osteoporosis and sarcopenia demonstrate the simultaneous dependency of muscle and bone tissue quantity [2,5,6]. Therefore, it has long been postulated that the regulation of bone mass was solely due to mechanical adaptations to the neighboring muscle volume and its activity level.

Recently, this dogma has been challenged with the description of the myostatin-null mice. Myostatin (growth differentiation factor-8) is highly expressed in muscles and suppresses muscle growth. Mutations of the myostatin gene in cattle and mice result in a phenotype of large muscle mass [7,8]. However, despite the impressive increase of muscle mass and overall muscle strength in the myostatin-null mice, the bone parameters of the femur are not majorly affected [9]. Such “uncoupling” of muscle and bone is also found in transgenic mice overexpressing interleukin-15 (IL-15) in the skeletal muscle [10]. Here, mice present with increased bone mass but similar lean mass compared to control mice. Further research on this phenotype suggests the increased bone mass not being related to alterations in biomechanics [11], and an additional regulatory circuit to permit the musculoskeletal interaction was suggested. The hypothesis of the existence of a biochemical communication of both tissues was further strengthened by a fracture healing studies. In a murine model of open tibial fractures, healing was improved if the wound was covered by muscle flaps suggesting that soluble factors/myokines contribute to bone healing [12].

## 2. Biomechanical Regulation of Muscle and Bone

The biomechanical relationship of bone and muscle has been well described historically. Skeletal muscles attach to bone and facilitate motion via a muscular contraction. Therefore, muscles expose bone to different kinds of mechanical stimuli depending on the muscular activity (isometric, static, plyometric, concentric, eccentric, low/high frequency, etc.). The attachment site of muscle is in local proximity to the axes of motion, which results in small lever arms. As a result, large forces have to be generated by muscle and are transmitted to the skeleton to produce the motion-required torque at the end of the lever arm (bone) [3]. It has then been proposed that such muscle-derived forces are the primary source of mechanical loading that generate the strain in bone [13].

One piece of evidence that muscle-generated forces are affecting bone directly comes from studies on the embryonal development of the muscular–skeletal unit. During this, muscles exert forces on bone to facilitate the formation of a mechanically optimal bone shape, which is able to resist deformation later in life. In mice paralyzed due to muscular dysgenesis *in utero*, the long bone diaphysis has acquired a round shape that is less likely to resist mechanical loading [14]. This has been also observed by Rot-Nikcevic I et al. [15] in amyogenic mice, lacking striated muscles.

Further support for the notion that muscle forces influence bone directly is seen during the acquisition of peak bone mass with pre-pubertal growth. Here, exercise was shown to have significant effects on bone mass. The beneficial effects of physical activity are also seen later in life, even if to a lesser extent [16].

The biomechanical coupling in the musculoskeletal unit is explained by the mechanostat theory, which states that bone adjusts its mass and architecture to experience strains within a physiological window [17]. Strains greater than this window will induce bone formation, while lower strains will lead to bone resorption. In addition to load transmission between muscle and bone, the two tissues show codependent hypertrophic or hypotrophic adaptations. Physical activity increases both muscle and bone mass [5], while aging or disuse leads to loss of mass in both organs [6].

Several animal models have been developed to help understand the codependency of both tissues; however, each of them is based on a different mechanism leading to slightly different results. Neurectomy, where a small piece of the sciatic nerve is removed using surgical intervention, is a common animal model to induce osteopenia and sarcopenia. It simulates a spinal cord injury in humans; here, complete or partial muscle paralysis is achieved and a rapid bone loss is observed. Despite the desired bone loss, this model does not solely dissect the mechanistic link between the dependency of muscle and bone, as bone may be influenced by neurological changes on its own [3,18,19]. Other alternatives are tenotomy, cast-induced immobilization, tail suspension, and intramuscular injection of botulinum toxin (Botox). All these techniques lead to muscle and bone loss, however each of them have some disadvantages. For example, with tail suspension, muscles are still able to contract while the hind limbs are in suspension, and with Botox-induced muscle paralysis muscle function is impaired but also the neuromuscular proprioceptive signaling. In addition, it has been reported that Botox does not solely affect muscle prior to affecting bone [20,21], however, in the situation of unloading, muscle atrophy does precede the loss of bone [22]. With the currently available findings it is possible that signaling pathways responsible for affecting morphology and function of muscle and bone are timely inseparable or are timely consecutive [3].

To demonstrate a direct relationship between muscle and bone, Botox injection has been coupled with tail suspension in a study by Warden SJ et al. [23]. With this approach, the authors aimed to obtain mechanical loading near zero level. The combined treatment did present with greater detrimental effects on the skeleton than tail suspension or Botox injection alone, demonstrating that Botox-induced muscle inhibition has skeletal effects over and above any effect it has in altering gravitational loading, suggesting that muscle has a direct effect on bone. However, from a disease point of view, sarcopenia does not fully account for the osteoporotic phenotype and osteoporosis does not fully account for sarcopenia, at least based on mass measures alone. This may be because bone quality and muscle function are better measures to reflect the basis of these diseases [24], or that, in addition to the biomechanical coupling of both tissues, a biochemical musculoskeletal interaction is taking place.

## 3. Biochemical Communication between Muscle and Bone: Muscle and Bone as Endocrine Organs

The endocrine relationship between muscle and bone is far less understood, however, increasing amounts of data have been accumulating in the past years making a strong case for the endocrine nature of both tissues (see Table 1 and Table 2). It is now well known that, following exercise, muscles secrete factors into the circulation that have effects on other tissues. Nowadays, these factors are known as “myokines” (Table 1).

Interleukin 6 (IL-6) was among the first myokines identified; it is produced in large amounts during exercise [25] by cells in type II muscle fibers [26]. IL-6 from muscle regulates satellite cell (muscle stem cells) differentiation to mediate skeletal hypertrophy [27]. Muscle secreted IL-6 not only exerts paracrine effects but also endocrine effects acting on distant organs, i.e., the liver and the adipose tissue. IL-6 null mice develop early mature-onset obesity [28]. Other interleukins have been documented since, such as IL-5, IL-7, and IL-8, which stimulates angiogenesis [29], Brain-Derived Neutrophic Factor (BDNF) is highly expressed in the brain, serum and skeletal muscle after exercise [30,31,32]. BDNF is involved in exercise-induced skeletal muscle regeneration [31] and fat oxidation [32]. Ciliary Neurotrophic Factor (CNTF) is a myokine inducing the suppression of bone formation at the periosteum. Muscle-derived IL-15 works to reduce adiposity and mice expressing high levels of IL-15 show increased bone mineral content [10].

Muscles secrete myostatin (growth differentiation factor-8, GDF-8), a member of the tumor growth factor-β family. Myostatin is a potent inhibitor of skeletal muscle cell proliferation and growth [33]. Myostatin binds to the activin receptor type II (ACVR2B) on muscle cells resulting in the intracellular phosphorylation of Smads 2 and 3, the aggregation with Smad 4 and the nuclear translocation to activate target genes [34] (for review, see Joulia-Ekaza [35]).

Irisin is a hormone-like molecule produced by muscle post exercise. Irisin is produced by the cleavage of the membrane protein Fndc5 under the regulation of PCI1α (PPARγ coactivator-1α). It is capable of “browning” certain white adipose tissues *in vitro* and *in vivo*, increases energy expenditure and improves glucose tolerance of high fat fed mice [36].

Until recent years, bone was not considered an endocrine organ, but rather as an endocrine targeted tissue that responds to hormones like parathyroid hormone (PTH) and sex steroids. However, increasing data demonstrates that bone produces factors now referred as “osteokines” that have effects on other tissues such as muscle, liver, kidneys and pancreas (Table 2).

Probably the first discovered hormone-like “osteokine” secreted by bone cells (osteocytes) was Fibroblast Growth Factor 23 (FGF23) [37]. Mutation in FGF23 is the cause of Autosomal Dominant Hypophosphatemic Rickets (ADHR). FGF23 and PTH might work together to regulate phosphate metabolism. FGF23 is known to act on the intestine and the kidney by downregulating the expression of sodium/phosphate co-transporters responsible to absorb and reabsorb phosphate [38,39,40]. Elevated levels of FGF-23 could play a role in cardiac hypertrophy, which suggests more widespread actions of this molecule [41].

Osteocalcin, or Bone Gamma-Carboxyglutamate Protein (BGLAP), is a secreted protein produced mainly by osteoblasts. It is bound to the bone extracellular matrix but has been found in the plasma with higher levels of expression at the fetal stage (in fetal calves) as compared to adulthood (in adult cows) [42]. Osteocalcin^−/−^ mice show decreased β-cell proliferation, insulin secretion and sensitivity [43], suggesting a regulatory role in glucose metabolism.

Sclerostin is a protein mainly secreted by osteocytes. In bone, sclerostin binds to the second or third β-propeller of the Wnt/LRP/Frizzle tri-molecular complex inhibiting the activation of the Wnt/β-catenin pathway [44], an important regulator of bone and muscle mass during development, growth and adaptation. The Wnt/β-catenin pathway may play a huge role in the endocrine crosstalk between bone (osteocyte) and distant organs as sclerostin is a secreted protein and it can be detected in plasma. However, it remains controversial as to whether high levels of sclerostin in the plasma can be correlated with increased facture risk [45,46].

Bone is also known to secrete factors like Dentin Matrix Protein 1 (DMP1) [47], matrix extracellular phosphoglycoprotein (MEPE), and phosphate-regulating gene with homologies to endopeptidases on the X chromosome (PHEX), all of which are involved in phosphate metabolism. Dmp1 knockout mice present with increased levels of FGF23 [48].

**Figure 2:**
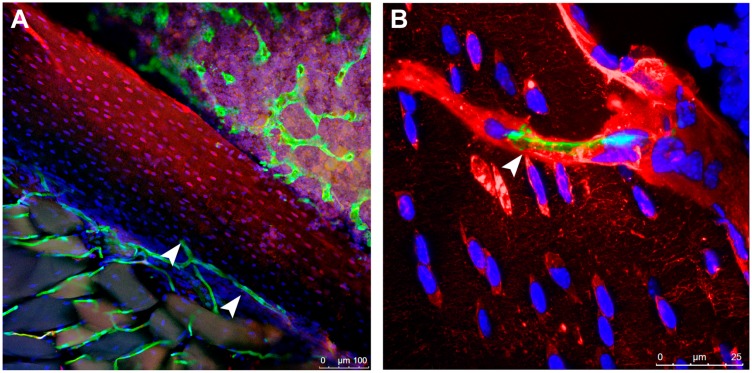
Role of vessels in the muscle–bone crosstalk: (**A**) Presence of vessels coming from the skeletal muscle in bone (white arrowheads). The vessels are stained in green, in transgenic mice model Flk1-GFP, where the green fluorescent protein is driven by a promoter targeting a receptor of VEGF-A [50]. Magnification: 10×; (**B**) physical connection between osteocytes in the femur (stained in red by a Dextran-lysine fixable stain) and a vessel, in green (Flk1-GFP mice) (white arrowhead). Magnification: 63×. Scale bars represent: 100 μm (**A**); and 25 μm (**B**).

In addition, bone is a source for growth factors like insulin-like growth factors (IGFs), transforming growth factor-beta (TGF beta) and bone morphogenetic proteins (BMPs) [49]. IGFs, TGF beta and BMPs are produced by osteoblasts and other bone cells and affect osteoblast proliferation and differentiation. Growth factors are incorporated in the mineralized bone matrix and retain their activity when extracted from bone during osteoclast-dependent bone resorption. These factors can be found in the circulation, reaching the blood system via the connection of the osteocyte lacuno-canalicular system with vessels in bone (Figure 2).

In recent years, researchers are studying in more detail the possibility of a bone–muscle crosstalk that is the actions of muscle-derived factors on bone and bone derived-factors on muscle. This type of communication appears to act in addition to the biomechanical interaction described in the previous section. The endocrine crosstalk and in particular the crosstalk via myokines and osteokines hold the potential for improving the mechanistic understanding of tissue functions within the musculoskeletal unit.

## 4. Muscle Secreted Factors Have Effects on Bone Tissue

Among the many factors produced by skeletal muscle, some affect bone cell (Table 1). Mice with elevated circulating levels of IL-15 have increased bone mineral content [10]. Another interleukin, IL-6 is produced by myotubes and can promote osteoclastogenesis *in vitro* [51]. Under physiological conditions, osteoblasts and osteoclasts also produce IL-6. Cells of the osteoblast lineage respond to several members of the IL-6 family (including LIF) by expressing RANK ligand (RANKL). The interaction of RANKL with RANK on osteoclast precursors induces the formation of mature bone-resorbing osteoclasts. At the same time, osteoclasts produce Cardiotrophin-1 (CT-1) which stimulates bone formation and suppresses adipogenesis [52] (for a more detailed review, see Sims et al. [53]). RANKL and osteoprotegerin (OPG), as known regulators of osteoclastogenesis, are produced by myocytes among other cell types [54].

Irisin, as described above, is secreted by muscle during exercise. It was first identified as a regulator of energy expenditure in white adipose tissue [55], but recently irisin was found to induce osteoblast differentiation *in vitro* [56] exerting anabolic effects on cortical bone [57]. Treatment of mice with irisin improves bone mineral density and bone strength [57]. Moreover, injection of recombinant irisin to mice exposed to hind-limb suspension prevents bone loss typically observed in this animal model [58].

Myostatin negatively regulates bone function. In *in vitro* studies, more bone marrow-derived mesenchymal stem cells (MSC) obtained from myostatin-deficient mice differentiated into osteoblasts than did bone marrow-derived MSC obtained from control animals [59]. Exercised myostatin-deficient mice had increased gain of bone strength as compared to control group, suggesting that myostatin controls bone response to loading. Myostatin enhances *in vitro* osteoclast formation by inducing osteoclast-related genes [60].

There may be more unreported/undiscovered myokines having a positive effect on bone cells. We recently reported that “unknown” secreted factors from C2C12 myotubes but not myoblasts, maintain the viability of MLOY4 osteocytes, when treated with dexamethasone [61]. In addition, *ex vivo* electrically stimulated skeletal muscles protected osteocytes against glucocorticoid-induced cell death, showing that muscle contraction induces the secretion of myokines that are osteo-protective.

The factors secreted by muscle may vary depending on muscle activity (eccentric and concentric contraction), disuse, aging or damage (traumatic injury) [11]. This has been reported by Juffer et al. [51] and by Mera et al. [62], and reviewed by Hamrick [11].

## 5. Bone Secreted Factors Have Effects on Muscle Tissue

The osteocyte network encompasses a global signaling network within bone. Osteocytes send signals to other osteocytes, osteoblasts and osteoclasts, as well as to their precursors to orchestrate bone remodeling. Bone responds to differential mechanical loading by regulating the production of the osteocyte factor sclerostin [63]. Sclerostin is a negative regulator of the anabolic Wnt/β-catenin pathway. This pathway regulates bone mass and crosstalks with the prostaglandin pathway, thereby decreasing the production of negative regulators of the pathway (Dkk1 and sclerostin) with anabolic loading [64]. It has been shown that two products produced by osteocytes in response to shear stress, prostaglandin E2 and Wnt 3a, support myogenesis and muscle function [65,66] (Table 2).

Recently, osteocalcin was found to affect muscle tissue [67]. This has been observed by G Karsenty’s group who showed that delivery of osteocalcin prior to exercise increases the exercise-capacity of young mice and restores aerobic endurance in old mice [62,68]. Osteocalcin even increased muscle mass in old mice [68,69].

## 6. Common Mechanisms Influencing Bone and Muscle Mass

Heritability studies have estimated that between 40% and 80% of all skeletal phenotypes are due to genetic determinants. The same has been reported for muscular traits [70,71]. Given the high degree of genetic influences underlying bone and muscle function and the coupled tissue growth and development, shared genetic components seem probable.

Bivariate Genome Wide Association Studies (GWAS) have recently identified pleiotropic candidate genes, single nucleotide polymorphisms and regions associated with traits in both bone and muscle. These studies produced a list of potential bone–muscle pleiotropic genes that do require validation experiments. Some genes of interests are *METTL21C* and *MEF2C*. The *MEF2C* gene encodes a transcription factor (myocyte enhancer factor 2C) that is involved in cardiac and skeletal muscle development and marks myogenic cells in the somites during development [72]. Lately, mice with a deletion of *Mef2c* in osteocytes were shown to have increased bone mineral density through a mechanism involving reduced *Sost* (gene product of sclerostin) expression and reduced osteoclastogenesis [73]. These data suggest a role of *MEF2C* both in skeletal development and in the regulation of bone mass.

Secreted factors such as activins and pro-inflammatory cytokines represent potential common mechanisms linking bone and muscle. However, little is known regarding the action of these factors on muscle and bone mass, in particular during aging [74]. Activin signaling has been found to mediate the cytokine effect on myoblast differentiation, as activin A has to be upregulated for the anti-differentiation of the cytokines to be possible, through the ActRII/ALK/SMAD pathway [75]. Osteoactivin is a glycoprotein that is highly expressed during osteoblast differentiation. It is important for osteoblast differentiation and mineralization *in vitro* and regulates osteoblastogenesis *in vivo* [76]. It is increased during bone regeneration, and positively regulates fracture healing. Osteoactivin is also expressed by osteoclasts and stimulates osteoclast activity, differentiation and bone resorption [77]. With regard to the muscle–bone crosstalk, osteoactivin is expressed by muscles, upregulated during space flight, denervation, tail suspension and is able to induce transdifferentiation of myoblasts into osteoblasts [78,79].

One of the factors potentially shared between muscle and bone is insulin-like growth factor 1 (IGF-1), an anabolic factor for both bone and muscle. However, the final proof of secretion of IGF-1 from one tissue specifically affecting the other is still lacking [80].

Several studies have suggested an association between osteoporosis, sarcopenia and vitamin D. However, the exact role of vitamin D in these pathologies has not yet been fully understood. Vitamin D deficiency is often associated with elevated levels of PTH causing bone resorption and muscle weakness [54,81].

Sex steroids (estrogens and testosterone) and glucocorticoids are known to affect both muscle and bone. While the first exert positive effects on tissue function [82], the latter are known to negatively affect the musculoskeletal unit [83,84].

Similar to IGF-1, fibroblast growth factor 2 (FGF2) is also present at the muscle–bone interface and periosteum (in mice). FGF2 is released by wounded over-loaded myofibers and induces a growth response in muscle tissue [85]. In ovariectomized mice, FGF2 administration induces bone formation [86], therefore, this molecule could be another muscle factor acting on bone.

## 7. Indirect Links

Muscle and bone are physically connected through tendons, ligaments, cartilage, and other connective tissues. All of these could also affect the muscle–bone crosstalk.

It has been shown that the periosteum, which is the fibrous membrane that physically separates bone and muscle tissues, is both a functional target for muscle and bone derived factors and a gatekeeper for fluid and solute exchange between bone and muscle [87,88]. *Ex vivo* experiments with fluorescent tracers of different molecular weight revealed that the periosteum is semi-permeable and possesses a cut-off size of approximately 40 kDa [89]. Myokines such as PGE2, IGF-1, IL-15 and FGF-2 satisfy this molecular weight cut-off, while other candidates of the bone–muscle crosstalk such as IL-6 and TGF-β are less likely to meet this criterion. Their penetration time across the periosteum is higher than their bioactive lifetime [89]. However, myokines secreted by muscle tissue may reach bone through the vasculature (Figure 2). The amount of the secretome and the factor polarity might affect the tissue-to-tissue transport. In addition, the muscular activity state seems to determine the amount of myokines released, as does age and disease state. *In vivo* experiments are needed i.e., utilizing fluorescently labeled myokines to confirm the transport to bone tissue and their inter-tissue activity.

## 8. Nervous System

The sympathetic nervous system has been shown to regulate bone mass. In particular, leptin signaling in the brain is responsible for skeletal changes without the need of a humoral signal [90]. Osteoblasts and osteoclasts express functional β2-adrenergic receptors, which if blocked lead to increased cancellous bone mass [90]. Similarly, neuropeptide Y receptors (Y1 and Y2) are related to bone homeostasis. Their deletion in transgenic mice has an anabolic effect on bone [91,92,93]. Other central pathways have been shown to regulate bone, such as the cannabinoid system, melanocortins and neuromedin U (for detailed review see Houweling P. et al. [94]).

Muscle contraction is primarily governed by the central and somatic systems, where an action potential from the CNS stimulates motor neurons which activate muscle fibers. Neuronal inputs are fundamental for muscle physiology and muscle contraction and are an important mechanism for the muscle–bone interaction. Bone tissue relies upon neuronal actions in the muscle for its growth and development [94]. The sympathetic nervous system is also playing a role in skeletal muscle. Synthetic β-adrenergic receptors agonists induce muscle hypertrophy and reduce skeletal muscle wasting and atrophy [95,96]. β2AR signaling is important in skeletal muscle growth, development and regeneration in healthy populations [97,98,99,100]. As leptin regulates cancellous bone formation via β2AR signaling in osteoblasts and osteoclasts, and β2AR signaling stimulates skeletal muscle growth in disease and in healthy populations, β2AR may provide a possible link for the production and regulation of both muscle and bone tissues [94]. Research has focused on genetic, paracrine and metabolic interactions but the neuronal signaling may be a mechanism by which muscle and bone are co-regulated.

In aging, a relationship between obesity and metabolic syndrome has been observed. Energy restriction and exercising induces changes in muscle and bone [74]. Exercise and fat loss favorably affect bone and muscle mass in overweight people [101,102]. Fat interacts with muscle and bone [103]. Brown fat is more desirable than white fat and fat mass can be modified e.g., by exercise. The sympathetic nervous system plays a role in the regulation of fat type, but negatively affects skeletal remodeling. Myokines such as irisin, but also “osteokines” such as sclerostin can increase the formation of beige fat, and therefore exert further effects on muscle and bone tissues as described above.

Another possible way to modify the muscle–bone crosstalk are macrophages. Muscles secrete factors that affect bone, while macrophages affect muscle. Macrophages belong to the same cell lineage as osteoclasts. They are derived from hematopoietic precursor cells that have the capacity to differentiate to macrophages or osteoclasts, even macrophages can differentiate into osteoclasts within a suitable microenvironment [104]. A specific type of macrophages in bone is called “osteomacs”, which reside among lining cells in both the endosteum and the periosteum, and regulate osteoblast function [105]. Macrophages present as two subtypes: M1 and M2. M1 macrophages release pro-inflammatory cytokines while M2 macrophages promote growth and regeneration of muscle [106]. A switch can occur between M1 and M2 macrophages during regeneration [107]. M2 macrophages are highly present in injured muscle and promote regeneration and aid satellite function. These cells are part of the muscle regeneration response to unloading [108].

## 9. Fracture Healing

The interaction of bone and muscle is important in the context of fracture healing. Little is known of the biochemical crosstalk between muscle and bone in fracture healing except that fractures heal better if covered with muscle flaps [12,109,110]. When the muscle tissue around the fracture is damaged, or muscle atrophy is present, fracture healing is significantly impaired. Botox injections to induce muscle paralysis showed lower healing response in a femoral fracture model in rats [111]. However, this effect could be due to decreased muscular contractions (biomechanic), or due to a decreased myokine secretome (biochemical).

In addition to these, myoblasts have the capacity to transdifferentiate into the osteoblast lineage. Myogenesis of C2C12 cells can be halted with BMP2 treatment and the osteoblastic phenotype can be induced [112]. In this line of evidence, a few studies have demonstrated that satellite cells (myoblast progenitors) play a role in bone repair [113,114]. They seem to replace or co-exist with the bone cells population in the case of unavailable stem cells from the bone marrow or the periosteum.

Another path of future exploration is the role of muscular vessels in bone repair, especially the potential link between endothelial cells and bone cells. A recent study from Prasadam I et al. showed that conditioned media from the MLO-Y4 osteocyte increased the proliferation, migration and tube-like formation of the endothelial cells, which was accompanied by the expression of angiogenic genes in the endothelial cells [115]. There could be a crosstalk between bone and muscle via vessels, especially in the case of fracture repair. However, the crosstalk between these three tissues remains to be further characterized.

## 10. Other Factors Affecting the Musculoskeletal Health—The Molecular Clock

Physiology and behavior are temporally coordinated into rhythms coinciding with the 24 h solar cycle. These circadian rhythms are underlined by a mechanism called the molecular clock. It comprises of a series of interconnected transcriptional–translational feedback loops [116]. This system functions to optimize the timing of cellular events in anticipating environmental changes, e.g., daylight and food availability. The mechanisms by which clocks in one tissue influence the physiology of another tissue has not been well studied. To date, only one study reports that skeletal muscle rhythms are important for the maintenance of bone health [117]. Another analysis utilizing microarray data has identified several myokines that significantly change expression following the skeletal muscle specific knock-out of *Bmal 1* (brain muscle arnt-like 1,, encoding the protein Aryl hydrocarbon receptor nuclear translocator-like protein 1), a non-redundant gene within the core feedback loop [118]. The mRNA expression of several myokines with a known effect on bone is altered in these mice [116]. Among the differentially expressed genes, muscle–bone-crosstalk mediators, e.g., *Fndc5*/*Irisin*, *Vegfa* (Vascular endothelial growth factor A), *Tgfb1* (Transforming growth factor beta-1), *Igfbp4* (insulin like growth factor binding protein 4), *Il15* (Interleukin-15), *Mstn* (myostatin) and *Igfbp5* (Insulin-like growth factor binding protein 5), were found.

Very few papers have investigated the role of the molecular clock in bone tissue function, making the mechanistic understanding of a crosstalk on this level from bone to muscle difficult at this time. We can note that the deletion of proprotein convertase *Mbtps1* gene (membrane bound transcription factor peptidase, site 1) in osteocytes stimulates soleus muscle regeneration, size and contractile force [119]. Many of the myogenic genes altered in this larger and functionally improved muscle were regulated by the circadian core transcriptional repressors DEC1 (Deleted In Esophageal Cancer 1) and DEC2 [120].

Exosomes and their microRNA cargos are other factors that could affect the muscle–bone crosstalk. They are reviewed by Cardozo and Graham [121].

## 11. Pharmacological Interventions

The treatment of osteoporosis aims to reduce fracture risk by increasing bone mass, or at least preventing further bone loss. Therapeutics therefore mainly act to: (i) inhibit bone resorption; or (ii) enhance bone formation. Exercise and lifestyle changes (nutrition) are also recommended for osteopenic/osteoporotic patients. Additionally, vitamin D and calcium supplementation are recommended to aid the prevention and treatment of osteoporosis. Among the anti-resorptive drugs (mainly acting to prevent bone resorption) are the group of bisphosphonates (alendronate and risedronate), selective estrogen receptor modulators (SERMs) (raloxifene), monoclonal antibodies (denosumab), calcitonin, estrogens, hormone replacement therapy and strontium ranelate [122]. Among the anabolic drugs, the list is shorter. PTH and its shorter peptide teriparatide are currently the only approved osteoanabolic drugs; however, the sclerostin antibodies (blosozumab and romosozumab) might come to the market soon. PTH or teriparatide, if given intermittently, increase bone formation by enhancing osteoblast activity and precursor recruitment. However, the long term treatment of these increases the risk of osteosarcoma, therefore, drug-holiday and a combination with other drugs is often advised (Table 3).

Overall, a long term fracture risk reduction with the existing strategies is hard to achieve [54]. While this is the major cause to continue the search for more effective drugs preventing the detrimental osteoporotic fracture, there are also several adverse effects with the available pharmacological drugs (Table 3).

Currently, no FDA-approved drugs are available to treat sarcopenia. Patients are advised to perform resistance-training exercise to re-gain muscle force and prevent falls. Exercise increases both muscle and bone mass. Bone responds in an anabolic manner to low impact high frequency exercise, while resistance exercise leads to muscle mass and force gains. However, these benefits decrease with age or disease. For patients who are unable to perform exercise, this option is not a possible approach to increase muscle mass. However, even with the potential upcoming myostatin inhibitors (bimagrumab, PINTA 745), there is a need for new pharmacological approaches to maintain or rebuild a healthy muscular system.

The development of drugs that target both bone and muscle tissues is ongoing. Clinical trials for several of the molecules mentioned above are currently undertaken. These include IGF-1, which has showed that it increased BMD and is reviewed by Lindsey RC et al. [123]. Osteocalcin, irisin, myostatin could be also targets for future trials in order to become drugs for both osteoporosis and sarcopenia treatments (for detailed review on these, see [124]).

Studying the effects of these future drugs on both bone and muscle, investigating the mechanisms of diseases that present with uncoupling of bone and muscle, e.g., osteogenesis imperfecta, or diseases that are characterized with muscle weakness without muscle myopathy will aid the mechanistic understanding of the muscle–bone crosstalk [125].

With such investigations, researchers could answer these pressuring questions: Is the muscle or bone secretome changing with aging? What happens in pathologies e.g., cancer that affects the musculoskeletal system? What are the factors that are increased or decreased with pathologies? What happens in the case of muscle or bone regeneration? Do the satellite cells produce anabolic factors for bone [126]?

## 12. Conclusions

Over the years, researchers have adapted their knowledge on the musculoskeletal interaction. The biomechanical interaction of both tissues has received great attention, as has the molecular understanding of each tissue function. More recently, the biochemical communication of both tissues has gained great interest. Together, these findings will create a deeper understanding of tissue functions within the musculoskeletal unit and will aid the development of novel therapeutics. The muscle–bone crosstalk is complex. Additionally, adjacent tissues may affect this crosstalk. Finally, the crosstalk seems to be a function of aging, disease state and activity level.

## Figures and Tables

**Table 1:** Myokines known to date, and their effects on bone.

Molecule	Effect on Bone
Myostatin	Promotes osteoclastogenesis
Irisin	Promotes osteoblast differentiation
Insulin-like Growth Factor (IGF-1)	Increases ability of osteoblast to deposit bone
Basic Fibroblast Growth Factor-2 (FGF-2)	Promotes osteoblastogenesis
Interleukin-6 (IL-6)	Increases osteoclastogenesis by promoting RANKL secretion by osteoblasts
Interleukin-15 (IL-15)	Promotes osteoblast capacity to deposit mineral matrix
Interleukin-5 (IL-5)	Not determined
Interleukin-7 (IL-7)	Inhibitor of osteoclastogenesis in bone marrow cultures
Interleukin-8 (IL-8)	Not determined
Brain-Derived Neurotrophic Factor (BDNF)	Regulates expression and secretion of VEGF from osteoblasts
Ciliary Neurotrophic Factor (CNTF)	Suppresses osteoblast differentiation *in vitro*
Follistatin-like protein 1	Not determined
Decorin	Promotes bone matrix formation and calcium deposition
Osteoglycin (OGN)	Increases alkaline phosphatase, type I collagen and osteocalcin

RANKL: Receptor Activator of Nuclear factor Kappa-B Ligand; VEGF: Vascular Endothelial Growth Factor

**Table 2:** Osteokines and the effects on muscle that are known.

Molecule	Effect on Muscle
Osteocalcin or Bone Gamma-Carboxyglutamate Protein (BGLAP)	Increases insulin sensitivity, promotes protein synthesis in myotubes
Fibroblast Growth Factor (FGF23)	Not determined
Sclerostin	Not determined
Dentin Matrix Protein-1 (DMP-1)	Not determined
Matrix Extracellular Phosphoglycoprotein (MEPE)	Not determined
Phosphate-regulating gene with Homologies to Endopeptidases on the X chromosome (PHEX)	Not determined
Receptor Activator of Nuclear Factor-kappa B Ligand (RANKL)	Not determined
Prostaglandin E2 (PEG2)	Promotes proliferation of myoblasts
WNT-3a	Enhances muscle ability to contract

**Table 3:** Drugs currently available to treat osteoporosis.

Generic Name	Commercial Name	Approved by FDA	Route of Administration	Effect on Bone	Mechanism of Action	Major Side Effect
**Alendronate**	Fosamax, Binosto	Yes	Oral (daily or weekly)	Anti-resorptive	Inhibits osteoclast formation and activity	Atypical subtrochanteric and diaphyseal femoral fractures
**Risedronate**	Actonel Atelvia	Yes	Oral, long-lasting tablet (one tablet weekly or on tablet monthly or one tablet per day for 2 consecutive days each month)	Anti-resorptive	Inhibits osteoclast activity	Atypical subtrochanteric and diaphyseal femoral fractures
**Ibandronate**	Boniva	Yes	Intravenous injection once every three months	Anti-resorptive	Inhibits osteoclast activity	Atypical subtrochanteric and diaphyseal femoral fractures
**Zoledronic acid**	Reclast	Yes	Intravenous injection once a year	Anti-resorptive	Inhibits release of acid by osteoclasts	Atypical subtrochanteric and diaphyseal femoral fractures
**SERM (Raloxifene)**	Evista, Keoxifene	Yes	Oral (daily)	Anabolic	Binds to estrogen receptors (Estrogen agonist)	Might develop blood clot in lung or lungs
**Denosumab**	Prolia, Xgeva	Yes	Subcutaneous injection (once every 6 months for osteoporosis treatment)	Anti-resorptive	Binds to RANKL	Femoral bone fracture
**Estrogens**	Amnestrogen, Cenestin, Enjuvia, Estrace, Estratab, Evex, Femogen, Menest, Ogen Tablets, Ortho-est, Premarin	Yes	Oral (daily)	Anabolic/Anti-resorptive	Binds to DNA activating targeted genes. Promotes osteoclast apoptosis	Increase risk to develop endometrial cancer
**Hormone replacement therapy**	Activella, Angeliq, FemHRT, Jinteli, Mimvey, Prefest, Premphase, Prempro	Yes	Oral (daily)	Anabolic	Binds to DNA activating targeted genes. Promotes osteoclast apoptosis	May increase the risk of heart attack, stroke, breast cancer, and blood clots in the lungs and legs
**PTH (Teriparatide)**	Forteo	Yes	Subcutaneous injection daily for up to 2 years	Anabolic	Increases osteoblast activity and recruitment	Osteosarcoma
**Strontium ranelate**	Protelos, Osseor	Alternative use only	Oral (daily)	Anabolic/Anti-resorptive	May induce osteoblast proliferation and osteoclast apoptosis	Heart problems, blood clots
**Blosozumab**		No (Phase III Clinical trials)	Subcutaneous injection	Anabolic	Inhibits Sclerostin (activates Wnt/b-catenin pathway	Increase cardiovascular events
**Romosozumab**	Evenity	No (Phase III of Clinical trials)	Subcutaneous injection	Anabolic	Inhibits Sclerostin (activates Wnt/b-catenin pathway	Increase cardiovascular events
**Abaloparatide**	Tymlos	Yes	subcutaneous injection once daily	Anabolic	Parathyroid hormone-related peptide analogue	Increase incidence of osteosarcoma (in mice)
**Odonacatib**		No		Anti-resorptive	Cathepsin-K antagonist	Elevated incidence of stroke

## References

[1] Fabbri E., Zoli M., Gonzalez-Freire M., Salive M.E., Studenski S.A., Ferrucci L. (2015). Aging and Multimorbidity: New Tasks, Priorities, and Frontiers for Integrated Gerontological and Clinical Research. J. Am. Med. Dir. Assoc..

[2] Mitchell W.K., Williams J., Atherton P., Larvin M., Lund J., Narici M. (2012). Sarcopenia, Dynapenia, and the Impact of Advancing Age on Human Skeletal Muscle Size and Strength; a Quantitative Review. Front. Physiol..

[3] Avin K.G., Bloomfield S.A., Gross T.S., Warden S.J. (2015). Biomechanical Aspects of the Muscle-Bone Interaction. Curr. Osteoporos. Rep..

[4] Schiessl H., Frost H.M., Jee W.S.S. (1998). Estrogen and Bone-Muscle Strength and Mass Relationships. Bone.

[5] Ducher G., Courteix D., Même S., Magni C., Viala J.F., Benhamou C.L. (2005). Bone geometry in response to long-term tennis playing and its relationship with muscle volume: A quantitative magnetic resonance imaging study in tennis players. Bone.

[6] Reginster J.-Y., Beaudart C., Buckinx F., Bruyère O. (2016). Osteoporosis and sarcopenia: Two diseases or one?. Curr. Opin. Clin. Nutr. Metab. Care.

[7] McPherron A.C., Lawler A.M., Lee S.J. (1997). Regulation of skeletal muscle mass in mice by a new TGF-beta superfamily member. Nature.

[8] McPherron A.C., Lee S.-J. (1997). Double muscling in cattle due to mutations in the myostatin gene. Proc. Natl. Acad. Sci. USA.

[9] Hamrick M.W., McPherron A.C., Lovejoy C.O., Hudson J. (2000). Femoral morphology and cross-sectional geometry of adult myostatin-deficient mice. Bone.

[10] Quinn L.S., Anderson B.G., Strait-Bodey L., Stroud A.M., Argilés J.M. (2009). Oversecretion of interleukin-15 from skeletal muscle reduces adiposity. Am. J. Physiol. Endocrinol. Metab..

[11] Hamrick M.W. (2012). The skeletal muscle secretome: An emerging player in muscle–bone crosstalk. BoneKEy Rep..

[12] Harry L.E., Sandison A., Paleolog E.M., Hansen U., Pearse M.F., Nanchahal J. (2008). Comparison of the healing of open tibial fractures covered with either muscle or fasciocutaneous tissue in a murine model. J. Orthop. Res..

[13] Frost H.M. (2000). Muscle, bone, and the Utah paradigm: A 1999 overview. Med. Sci. Sports Exerc..

[14] Sharir A., Stern T., Rot C., Shahar R., Zelzer E. (2011). Muscle force regulates bone shaping for optimal load-bearing capacity during embryogenesis. Development.

[15] Rot-Nikcevic I., Reddy T., Downing K.J., Belliveau A.C., Hallgrímsson B., Hall B.K., Kablar B. (2006). Myf5^−/−^:MyoD^−/−^ amyogenic fetuses reveal the importance of early contraction and static loading by striated muscle in mouse skeletogenesis. Dev. Genes Evol..

[16] Gunter K.B., Almstedt H.C., Janz K.F. (2012). Physical Activity in Childhood May Be the Key to Optimizing Lifespan Skeletal Health. Exerc. Sport Sci. Rev..

[17] Frost H.M. (2003). Bone’s mechanostat: A 2003 update. Anat. Rec. A Discov. Mol. Cell. Evol. Biol..

[18] Dudley-Javoroski S., Shields R.K. (2008). Muscle and bone plasticity after spinal cord injury: Review of adaptations to disuse and to electrical muscle stimulation. J. Rehabil. Res. Dev..

[19] Elefteriou F. (2008). Regulation of bone remodeling by the central and peripheral nervous system. Arch. Biochem. Biophys..

[20] Poliachik S.L., Bain S.D., Threet D., Huber P., Gross T.S. (2010). Transient muscle paralysis disrupts bone homeostasis by rapid degradation of bone morphology. Bone.

[21] Manske S.L., Boyd S.K., Zernicke R.F. (2010). Muscle and bone follow similar temporal patterns of recovery from muscle-induced disuse due to botulinum toxin injection. Bone.

[22] Lloyd S.A., Lang C.H., Zhang Y., Paul E.M., Laufenberg L.J., Lewis G.S., Donahue H.J. (2014). Interdependence of Muscle Atrophy and Bone Loss Induced by Mechanical Unloading. J. Bone Miner. Res..

[23] Warden S.J., Galley M.R., Richard J.S., George L.A., Dirks R.C., Guildenbecher E.A., Judd A.M., Robling A.G., Fuchs R.K. (2013). Reduced gravitational loading does not account for the skeletal effect of botulinum toxin-induced muscle inhibition suggesting a direct effect of muscle on bone. Bone.

[24] Brotto M., Johnson M.L. (2014). Endocrine Crosstalk between Muscle and Bone. Curr. Osteoporos. Rep..

[25] Steensberg A., van Hall G., Osada T., Sacchetti M., Saltin B., Pedersen B.K. (2000). Production of interleukin-6 in contracting human skeletal muscles can account for the exercise-induced increase in plasma interleukin-6. J. Physiol..

[26] Hiscock N., Chan M.H.S., Bisucci T., Darby I.A., Febbraio M.A. (2004). Skeletal myocytes are a source of interleukin-6 mRNA expression and protein release during contraction: Evidence of fiber type specificity. FASEB J..

[27] Serrano A.L., Baeza-Raja B., Perdiguero E., Jardí M., Muñoz-Cánoves P. (2008). Interleukin-6 is an essential regulator of satellite cell-mediated skeletal muscle hypertrophy. Cell Metab..

[28] Wallenius V., Wallenius K., Ahrén B., Rudling M., Carlsten H., Dickson S.L., Ohlsson C., Jansson J.-O. (2002). Interleukin-6-deficient mice develop mature-onset obesity. Nat. Med..

[29] Pedersen B.K., Åkerström T.C.A., Nielsen A.R., Fischer C.P. (2007). Role of myokines in exercise and metabolism. J. Appl. Physiol..

[30] Cuppini R., Sartini S., Agostini D., Guescini M., Ambrogini P., Betti M., Bertini L., Vallasciani M., Stocchi V. (2007). Bdnf expression in rat skeletal muscle after acute or repeated exercise. Arch. Ital. Biol..

[31] Yu T., Chang Y., Gao X.L., Li H., Zhao P. (2017). Dynamic Expression and the Role of BDNF in Exercise-induced Skeletal Muscle Regeneration. Int. J. Sports Med..

[32] Matthews V.B., Aström M.-B., Chan M.H.S., Bruce C.R., Krabbe K.S., Prelovsek O., Akerström T., Yfanti C., Broholm C., Mortensen O.H. (2009). Brain-derived neurotrophic factor is produced by skeletal muscle cells in response to contraction and enhances fat oxidation via activation of AMP-activated protein kinase. Diabetologia.

[33] Allen D.L., Cleary A.S., Speaker K.J., Lindsay S.F., Uyenishi J., Reed J.M., Madden M.C., Mehan R.S. (2008). Myostatin, activin receptor IIb, and follistatin-like-3 gene expression are altered in adipose tissue and skeletal muscle of obese mice. Am. J. Physiol. Endocrinol. Metab..

[34] Langley B., Thomas M., Bishop A., Sharma M., Gilmour S., Kambadur R. (2002). Myostatin Inhibits Myoblast Differentiation by Down-regulating MyoD Expression. J. Biol. Chem..

[35] Joulia-Ekaza D., Cabello G. (2007). The myostatin gene: Physiology and pharmacological relevance. Curr. Opin. Pharmacol..

[36] Boström P., Wu J., Jedrychowski M.P., Korde A., Ye L., Lo J.C., Rasbach K.A., Boström E.A., Choi J.H., Long J.Z. (2012). A PGC1-α-dependent myokine that drives brown-fat-like development of white fat and thermogenesis. Nature.

[37] Liu S., Zhou J., Tang W., Jiang X., Rowe D.W., Quarles L.D. (2006). Pathogenic role of Fgf23 in Hyp mice. Am. J. Physiol. Endocrinol. Metab..

[38] Hu M.C., Shiizaki K., Kuro-o M., Moe O.W. (2013). Fibroblast Growth Factor 23 and Klotho: Physiology and Pathophysiology of an Endocrine Network of Mineral Metabolism. Annu. Rev. Physiol..

[39] Quarles L.D. (2012). Skeletal secretion of FGF-23 regulates phosphate and vitamin D metabolism. Nat. Rev. Endocrinol..

[40] Gattineni J., Bates C., Twombley K., Dwarakanath V., Robinson M.L., Goetz R., Mohammadi M., Baum M. (2009). FGF23 decreases renal NaPi-2a and NaPi-2c expression and induces hypophosphatemia *in vivo* predominantly via FGF receptor 1. Am. J. Physiol. Ren. Physiol..

[41] Faul C., Amaral A.P., Oskouei B., Hu M.-C., Sloan A., Isakova T., Gutiérrez O.M., Aguillon-Prada R., Lincoln J., Hare J.M. (2011). FGF23 induces left ventricular hypertrophy. J. Clin. Investig..

[42] Nishimoto S.K., Price P.A. (1979). Proof that the gamma-carboxyglutamic acid-containing bone protein is synthesized in calf bone. Comparative synthesis rate and effect of coumadin on synthesis. J. Biol. Chem..

[43] Lee N.K., Sowa H., Hinoi E., Ferron M., Ahn J.D., Confavreux C., Dacquin R., Mee P.J., McKee M.D., Jung D.Y. (2007). Endocrine regulation of energy metabolism by the skeleton. Cell.

[44] Balemans W., Piters E., Cleiren E., Ai M., Van Wesenbeeck L., Warman M.L., Van Hul W. (2008). The binding between sclerostin and LRP5 is altered by DKK1 and by high-bone mass LRP5 mutations. Calcif. Tissue Int..

[45] Clarke B.L., Drake M.T. (2013). Clinical utility of serum sclerostin measurements. BoneKEy Rep..

[46] Ardawi M.-S.M., Rouzi A.A., Al-Sibiani S.A., Al-Senani N.S., Qari M.H., Mousa S.A. (2012). High serum sclerostin predicts the occurrence of osteoporotic fractures in postmenopausal women: The Center of Excellence for Osteoporosis Research Study. J. Bone Miner. Res..

[47] Toyosawa S., Shintani S., Fujiwara T., Ooshima T., Sato A., Ijuhin N., Komori T. (2001). Dentin Matrix Protein 1 Is Predominantly Expressed in Chicken and Rat Osteocytes but not in Osteoblasts. J. Bone Miner. Res..

[48] Feng J.Q., Ward L.M., Liu S., Lu Y., Xie Y., Yuan B., Yu X., Rauch F., Davis S.I., Zhang S. (2006). Loss of DMP1 causes rickets and osteomalacia and identifies a role for osteocytes in mineral metabolism. Nat. Genet..

[49] Linkhart T.A., Mohan S., Baylink D.J. (1996). Growth factors for bone growth and repair: IGF, TGF beta and BMP. Bone.

[50] Ishitobi H., Matsumoto K., Azami T., Itoh F., Itoh S., Takahashi S., Ema M. (2010). Flk1-GFP BAC Tg mice: An animal model for the study of blood vessel development. Exp. Anim..

[51] Juffer P., Jaspers R.T., Klein-Nulend J., Bakker A.D. (2014). Mechanically Loaded Myotubes Affect Osteoclast Formation. Calcif. Tissue Int..

[52] Walker E.C., McGregor N.E., Poulton I.J., Pompolo S., Allan E.H., Quinn J.M., Gillespie M.T., Martin T.J., Sims N.A. (2008). Cardiotrophin-1 Is an Osteoclast-Derived Stimulus of Bone Formation Required for Normal Bone Remodeling. J. Bone Miner. Res..

[53] Sims N.A. (2016). Cell-specific paracrine actions of IL-6 family cytokines from bone, marrow and muscle that control bone formation and resorption. Int. J. Biochem. Cell Biol..

[54] Picca A., Calvani R., Manes-Gravina E., Spaziani L., Landi F., Bernabei R., Marzetti E. (2017). Bone-muscle crosstalk: Unraveling new therapeutic targets for osteoporosis. Curr. Pharm. Des..

[55] Colaianni G., Mongelli T., Colucci S., Cinti S., Grano M. (2016). Crosstalk Between Muscle and Bone Via the Muscle-Myokine Irisin. Curr. Osteoporos. Rep..

[56] Colaianni G., Cuscito C., Mongelli T., Oranger A., Mori G., Brunetti G., Colucci S., Cinti S., Grano M. (2014). Irisin Enhances Osteoblast Differentiation *In Vitro*. Int. J. Endocrinol..

[57] Colaianni G., Cuscito C., Mongelli T., Pignataro P., Buccoliero C., Liu P., Lu P., Sartini L., Di Comite M., Mori G. (2015). The myokine irisin increases cortical bone mass. Proc. Natl. Acad. Sci. USA.

[58] Colaianni G., Mongelli T., Cuscito C., Pignataro P., Lippo L., Spiro G., Notarnicola A., Severi I., Passeri G., Mori G. (2017). Irisin prevents and restores bone loss and muscle atrophy in hind-limb suspended mice. Sci. Rep..

[59] Hamrick M., Shi X., Zhang W., Pennington C., Thakore H., Haque M., Kang B., Isales C.M., Fulzele S., Wenger K. (2007). Loss of Myostatin (GDF8) Function Increases Osteogenic Differentiation of Bone Marrow-Derived Mesenchymal Stem Cells but the Osteogenic Effect is Ablated with Unloading. Bone.

[60] Dankbar B., Fennen M., Brunert D., Hayer S., Frank S., Wehmeyer C., Beckmann D., Paruzel P., Bertrand J., Redlich K. (2015). Myostatin is a direct regulator of osteoclast differentiation and its inhibition reduces inflammatory joint destruction in mice. Nat. Med..

[61] Jähn K., Lara-Castillo N., Brotto L., Mo C.L., Johnson M.L., Brotto M., Bonewald L.F. (2012). Skeletal Muscle Secreted Factors Prevent Glucocorticoid-Induced Osteocyte Apoptosis through Activation of B-Catenin. Eur. Cells Mater..

[62] Mera P., Laue K., Ferron M., Confavreux C., Wei J., Galán-Díez M., Lacampagne A., Mitchell S.J., Mattison J.A., Chen Y. (2016). Osteocalcin signaling in myofibers is necessary and sufficient for optimum adaptation to exercise. Cell Metab..

[63] Klein-Nulend J., Bonewald L., Bilezikian J.P., Raisz L.G. (2008). Principles of Bone Biology.

[64] Bonewald L.F., Johnson M.L. (2008). Osteocytes, Mechanosensing and Wnt Signaling. Bone.

[65] Huang J., Mo C., Bonewald L., Brotto M. Wnt3a potentiates myogenesis in C2C12 myoblasts through changes of signaling pathways including Wnt and NFκB. Proceedings of the ASBMR 2014 Annual Meeting.

[66] Mo C., Romero-Suarez S., Bonewald L., Johnson M., Brotto M. (2012). Prostaglandin E2: From clinical applications to its potential role in bone-muscle crosstalk and myogenic differentiation. Recent Pat. Biotechnol..

[67] Levinger I., Scott D., Nicholson G.C., Stuart A.L., Duque G., McCorquodale T., Herrmann M., Ebeling P.R., Sanders K.M. (2014). Undercarboxylated osteocalcin, muscle strength and indices of bone health in older women. Bone.

[68] Mera P., Laue K., Wei J., Berger J.M., Karsenty G. (2016). Osteocalcin is necessary and sufficient to maintain muscle mass in older mice. Mol. Metab..

[69] Karsenty G., Mera P. (2017). Molecular bases of the crosstalk between bone and muscle. Bone.

[70] Arden N.K., Spector T.D. (1997). Genetic Influences on Muscle Strength, Lean Body Mass, and Bone Mineral Density: A Twin Study. J. Bone Miner. Res..

[71] Prior S.J., Roth S.M., Wang X., Kammerer C., Miljkovic-Gacic I., Bunker C.H., Wheeler V.W., Patrick A.L., Zmuda J.M. (2007). Genetic and environmental influences on skeletal muscle phenotypes as a function of age and sex in large, multigenerational families of African heritage. J. Appl. Physiol..

[72] Edmondson D.G., Lyons G.E., Martin J.F., Olson E.N. (1994). Mef2 gene expression marks the cardiac and skeletal muscle lineages during mouse embryogenesis. Development.

[73] Kramer I., Baertschi S., Halleux C., Keller H., Kneissel M. (2012). Mef2c deletion in osteocytes results in increased bone mass. J. Bone Miner. Res..

[74] Bonewald L.F., Kiel D., Clemens T., Esser K., Orwoll E., O’Keefe R., Fielding R. (2013). Forum on Bone and Skeletal Muscle Interactions: Summary of the Proceedings of an ASBMR Workshop. J. Bone Miner. Res..

[75] Trendelenburg A.U., Meyer A., Jacobi C., Feige J.N., Glass D.J. (2012). TAK-1/p38/nNFκB signaling inhibits myoblast differentiation by increasing levels of Activin A. Skelet. Muscle.

[76] Abdelmagid S.M., Belcher J.Y., Moussa F.M., Lababidi S.L., Sondag G.R., Novak K.M., Sanyurah A.S., Frara N.A., Razmpour R., Del Carpio-Cano F.E. (2014). Mutation in Osteoactivin Decreases Bone Formation *In Vivo* and Osteoblast Differentiation *In Vitro*. Am. J. Pathol..

[77] Sheng M.H.-C., Wergedal J.E., Mohan S., Amoui M., Baylink D.J., Lau K.-H.W. (2012). Targeted Overexpression of Osteoactivin in Cells of Osteoclastic Lineage Promotes Osteoclastic Resorption and Bone Loss in Mice. PLoS ONE.

[78] Nikawa T., Ishidoh K., Hirasaka K., Ishihara I., Ikemoto M., Kano M., Kominami E., Nonaka I., Ogawa T., Adams G.R. (2004). Skeletal muscle gene expression in space-flown rats. FASEB J..

[79] Sondag G.R., Salihoglu S., Lababidi S.L., Crowder D.C., Moussa F.M., Abdelmagid S.M., Safadi F.F. (2014). Osteoactivin induces transdifferentiation of C2C12 myoblasts into osteoblasts. J. Cell. Physiol..

[80] Venken K., Movérare-Skrtic S., Kopchick J.J., Coschigano K.T., Ohlsson C., Boonen S., Bouillon R., Vanderschueren D. (2007). Impact of androgens, growth hormone, and IGF-I on bone and muscle in male mice during puberty. J. Bone Miner. Res..

[81] Walker R.P., Paloyan E., Gopalsami C. (2004). Symptoms in patients with primary hyperparathyroidism: Muscle weakness or sleepiness. Endocr. Pract..

[82] Carson J.A., Manolagas S.C. (2015). Effects of sex steroids on bones and muscles: Similarities, parallels, and putative interactions in health and disease. Bone.

[83] Ziegler R., Kasperk C. (1998). Glucocorticoid-induced osteoporosis: Prevention and treatment. Steroids.

[84] Weinstein R.S., Jilka R.L., Parfitt A.M., Manolagas S.C. (1998). Inhibition of osteoblastogenesis and promotion of apoptosis of osteoblasts and osteocytes by glucocorticoids. Potential mechanisms of their deleterious effects on bone. J. Clin. Investig..

[85] Clarke M.S., Feeback D.L. (1996). Mechanical load induces sarcoplasmic wounding and FGF release in differentiated human skeletal muscle cultures. FASEB J..

[86] Liang H., Pun S., Wronski T.J. (1999). Bone anabolic effects of basic fibroblast growth factor in ovariectomized rats. Endocrinology.

[87] Evans S.F., Chang H., Knothe Tate M.L. (2013). Elucidating Multiscale Periosteal Mechanobiology: A Key to Unlocking the Smart Properties and Regenerative Capacity of the Periosteum?. Tissue Eng. Part B Rev..

[88] Hamrick M.W., McNeil P.L., Patterson S.L. (2010). Role of muscle-derived growth factors in bone formation. J. Musculoskelet. Neuronal Interact..

[89] Lai X., Price C., Lu X. (Lucas), Wang L. (2014). Imaging and Quantifying Solute Transport across Periosteum: Implications for Muscle-Bone Crosstalk. Bone.

[90] Takeda S., Elefteriou F., Levasseur R., Liu X., Zhao L., Parker K.L., Armstrong D., Ducy P., Karsenty G. (2002). Leptin Regulates Bone Formation via the Sympathetic Nervous System. Cell.

[91] Baldock P.A., Sainsbury A., Couzens M., Enriquez R.F., Thomas G.P., Gardiner E.M., Herzog H. (2002). Hypothalamic Y2 receptors regulate bone formation. J. Clin. Investig..

[92] Baldock P.A., Allison S.J., Lundberg P., Lee N.J., Slack K., Lin E.-J.D., Enriquez R.F., McDonald M.M., Zhang L., During M.J. (2007). Novel Role of Y1 Receptors in the Coordinated Regulation of Bone and Energy Homeostasis. J. Biol. Chem..

[93] Lee N.J., Nguyen A.D., Enriquez R.F., Doyle K.L., Sainsbury A., Baldock P.A., Herzog H. (2011). Osteoblast specific Y1 receptor deletion enhances bone mass. Bone.

[94] Houweling P., Kulkarni R.N., Baldock P.A. (2015). Neuronal control of bone and muscle. Bone.

[95] Hinkle R.T., Hodge K.M.B., Cody D.B., Sheldon R.J., Kobilka B.K., Isfort R.J. (2002). Skeletal muscle hypertrophy and anti-atrophy effects of clenbuterol are mediated by the beta2-adrenergic receptor. Muscle Nerve.

[96] Joassard O.R., Durieux A.-C., Freyssenet D.G. (2013). β2-Adrenergic agonists and the treatment of skeletal muscle wasting disorders. Int. J. Biochem. Cell Biol..

[97] Lynch G.S., Ryall J.G. (2008). Role of β-Adrenoceptor Signaling in Skeletal Muscle: Implications for Muscle Wasting and Disease. Physiol. Rev..

[98] Downie D., Delday M.I., Maltin C.A., Sneddon A.A. (2008). Clenbuterol increases muscle fiber size and GATA-2 protein in rat skeletal muscle in utero. Mol. Reprod. Dev..

[99] Beitzel F., Sillence M.N., Lynch G.S. (2007). β-Adrenoceptor signaling in regenerating skeletal muscle after β-agonist administration. Am. J. Physiol. Endocrinol. Metab..

[100] Beitzel F., Gregorevic P., Ryall J.G., Plant D.R., Sillence M.N., Lynch G.S. (2004). β2-Adrenoceptor agonist fenoterol enhances functional repair of regenerating rat skeletal muscle after injury. J. Appl. Physiol..

[101] Shah K., Armamento-Villareal R., Parimi N., Chode S., Sinacore D.R., Hilton T.N., Napoli N., Qualls C., Villareal D.T. (2011). Exercise training in obese older adults prevents increase in bone turnover and attenuates decrease in hip BMD induced by weight loss despite decline in bone-active hormones. J. Bone Miner. Res..

[102] Villareal D.T., Chode S., Parimi N., Sinacore D.R., Hilton T., Armamento-Villareal R., Napoli N., Qualls C., Shah K. (2011). Weight loss, exercise, or both and physical function in obese older adults. N. Engl. J. Med..

[103] Bermeo S., Gunaratnam K., Duque G. (2014). Fat and bone interactions. Curr. Osteoporos. Rep..

[104] Udagawa N., Takahashi N., Akatsu T., Tanaka H., Sasaki T., Nishihara T., Koga T., Martin T.J., Suda T. (1990). Origin of osteoclasts: Mature monocytes and macrophages are capable of differentiating into osteoclasts under a suitable microenvironment prepared by bone marrow-derived stromal cells. Proc. Natl. Acad. Sci. USA.

[105] Chang M.K., Raggatt L.-J., Alexander K.A., Kuliwaba J.S., Fazzalari N.L., Schroder K., Maylin E.R., Ripoll V.M., Hume D.A., Pettit A.R. (2008). Osteal Tissue Macrophages Are Intercalated throughout Human and Mouse Bone Lining Tissues and Regulate Osteoblast Function *In Vitro* and *In Vivo*. J. Immunol..

[106] Tidball J.G., Villalta S.A. (2010). Regulatory interactions between muscle and the immune system during muscle regeneration. Am. J. Physiol. Regul. Integr. Comp. Physiol..

[107] Deng B., Wehling-Henricks M., Villalta S.A., Wang Y., Tidball J.G. (2012). IL-10 triggers changes in macrophage phenotype that promote muscle growth and regeneration. J. Immunol..

[108] Kohno S., Yamashita Y., Abe T., Hirasaka K., Oarada M., Ohno A., Teshima-Kondo S., Higashibata A., Choi I., Mills E.M. (2012). Unloading stress disturbs muscle regeneration through perturbed recruitment and function of macrophages. J. Appl. Physiol..

[109] Utvåg S.E., Iversen K.B., Grundnes O., Reikerås O. (2002). Poor muscle coverage delays fracture healing in rats. Acta Orthop. Scand..

[110] Stein H., Perren S.M., Cordey J., Kenwright J., Mosheiff R., Francis M.J.O. (2002). The muscle bed—A crucial factor for fracture healing: A physiological concept. Orthopedics.

[111] Hao Y., Ma Y., Wang X., Jin F., Ge S. (2012). Short-term muscle atrophy caused by botulinum toxin-A local injection impairs fracture healing in the rat femur. J. Orthop. Res..

[112] Katagiri T., Yamaguchi A., Komaki M., Abe E., Takahashi N., Ikeda T., Rosen V., Wozney J.M., Fujisawa-Sehara A., Suda T. (1994). Bone morphogenetic protein-2 converts the differentiation pathway of C2C12 myoblasts into the osteoblast lineage. J. Cell Biol..

[113] Schindeler A., Liu R., Little D.G. (2009). The contribution of different cell lineages to bone repair: Exploring a role for muscle stem cells. Differ. Res. Biol. Divers..

[114] Liu R., Schindeler A., Little D.G. (2010). The potential role of muscle in bone repair. J. Musculoskelet. Neuronal Interact..

[115] Prasadam I., Zhou Y., Du Z., Chen J., Crawford R., Xiao Y. (2014). Osteocyte-induced angiogenesis via VEGF-MAPK-dependent pathways in endothelial cells. Mol. Cell. Biochem..

[116] Riley L.A., Esser K.A. (2017). The Role of the Molecular Clock in Skeletal Muscle and What It Is Teaching Us about Muscle-Bone Crosstalk. Curr. Osteoporos. Rep..

[117] Schroder E.A., Harfmann B.D., Zhang X., Srikuea R., England J.H., Hodge B.A., Wen Y., Riley L.A., Yu Q., Christie A. (2015). Intrinsic muscle clock is necessary for musculoskeletal health. J. Physiol..

[118] Hodge B.A., Wen Y., Riley L.A., Zhang X., England J.H., Harfmann B.D., Schroder E.A., Esser K.A. (2015). The endogenous molecular clock orchestrates the temporal separation of substrate metabolism in skeletal muscle. Skelet. Muscle.

[119] Gorski J.P., Huffman N.T., Vallejo J., Brotto L., Chittur S.V., Breggia A., Stern A., Huang J., Mo C., Seidah N.G. (2016). Deletion of Mbtps1 (Pcsk8, S1p, Ski-1) Gene in Osteocytes Stimulates Soleus Muscle Regeneration and Increased Size and Contractile Force with Age. J. Biol. Chem..

[120] Gorski J.P., Price J.L. (2016). Bone muscle crosstalk targets muscle regeneration pathway regulated by core circadian transcriptional repressors DEC1 and DEC2. BoneKEy Rep..

[121] Cardozo C.P., Graham Z.A. (2017). Muscle-bone interactions: Movement in the field of mechano-humoral coupling of muscle and bone. Ann. N. Y. Acad. Sci..

[122] Bernabei R., Martone A.M., Ortolani E., Landi F., Marzetti E. (2014). Screening, diagnosis and treatment of osteoporosis: A brief review. Clin. Cases Miner. Bone Metab..

[123] Lindsey R.C., Mohan S. (2016). Skeletal effects of growth hormone and insulin-like growth factor-I therapy. Mol. Cell. Endocrinol..

[124] Kawai M., Mödder U.I., Khosla S., Rosen C.J. (2011). Emerging Therapeutic Opportunities for Skeletal Restoration. Nat. Rev. Drug Discov..

[125] Brotto M., Bonewald L. (2015). Bone and Muscle: Interactions beyond Mechanical. Bone.

[126] Abou-Khalil R., Yang F., Lieu S., Julien A., Perry J., Pereira C., Relaix F., Miclau T., Marcucio R., Colnot C. (2015). Role of muscle stem cells during skeletal regeneration. Stem Cells (Dayt. Ohio).

